# Cell Cycle-Dependent Expression of Dub3, Nanog and the p160 Family of Nuclear Receptor Coactivators (NCoAs) in Mouse Embryonic Stem Cells

**DOI:** 10.1371/journal.pone.0093663

**Published:** 2014-04-02

**Authors:** Siem van der Laan, Eleonora Golfetto, Jean-Marc Vanacker, Domenico Maiorano

**Affiliations:** 1 Genome Surveillance and Stability laboratory, Department “Molecular Bases of Human Diseases”, CNRS-UPR1142, Institute of Human Genetics, Montpellier, France; 2 Physiopathology of orphan nuclear receptors, Institut de Génomique Fonctionnelle de Lyon, Ecole Normale Supérieure de Lyon, Lyon, France; The University of Hong Kong, Hong Kong

## Abstract

Pluripotency of embryonic stem cells (ESC) is tightly regulated by a network of transcription factors among which the estrogen-related receptor β (Esrrb). Esrrb contributes to the relaxation of the G1 to S-phase (G1/S) checkpoint in mouse ESCs by transcriptional control of the deubiquitylase Dub3 gene, contributing to Cdc25A persistence after DNA damage. We show that in mESCs, Dub3 gene expression is cell cycle regulated and is maximal prior G1/S transition. In addition, following UV-induced DNA damage in G1, Dub3 expression markedly increases in S-phase also suggesting a role in checkpoint recovery. Unexpectedly, we also observed cell cycle-regulation of Nanog expression, and not Oct4, reaching high levels prior to G1/S transition, finely mirroring Cyclin E1 fluctuations. Curiously, while Esrrb showed only limited cell-cycle oscillations, transcript levels of the p160 family of nuclear receptor coactivators (NCoAs) displayed strong cell cycle-dependent fluctuations. Since NCoAs function in concert with Esrrb in transcriptional activation, we focussed on NCoA1 whose levels specifically increase prior onset of Dub3 transcription. Using a reporter assay, we show that NCoA1 potentiates Esrrb-mediated transcription of Dub3 and we present evidence of protein interaction between the SRC1 splice variant NCoA1 and Esrrb. Finally, we show a differential developmental regulation of all members of the p160 family during neural conversion of mESCs. These findings suggest that in mouse ESCs, changes in the relative concentration of a coactivator at a given cell cycle phase, may contribute to modulation of the transcriptional activity of the core transcription factors of the pluripotent network and be implicated in cell fate decisions upon onset of differentiation.

## Introduction

Developmental transitions during very early embryogenesis are characterized by major rearrangements of the cell cycle [1], [2], [3]. Embryonic stem cells (ESCs) constitute a unique model for studying developmental processes since these cells have the unique feature of being pluripotent, and as such they can give rise to all cell lineages of the three primary germ layers upon differentiation [4]. The cell cycle of ESCs is uncommonly rapid as compared to a wide range of somatic cells due to shorter G1 and G2 gap phases, resulting in a characteristic high proportion of cells in S-phase. Interestingly, very recent data indicate that cell fate decisions are intimately linked to the cell cycle and in particular to the length of the G1-phase [5], [6]. Indeed, ESCs have a relaxed checkpoint at the G1/S transition, due to persistent abundance of Cdc25A, a phosphatase that by controlling the activity of CDKs (Cycle Dependent Kinase) regulates cell cycle transitions. Persistence of Cdc25A in G1 leads to constitutive CDK2 dephosphorylation so that the length of the G1 phase remains unaffected, even after DNA damage, thereby ensuring that mESCs remain pluripotent [6]. Cdc25A protein levels are tightly regulated through the cell cycle of somatic cells, and its turnover is the result of the opposite activities of the Dub3 deubiquitylase [7] and of the two ubiquitin ligase complexes, APC/C^Cdh1^ and SCF^βTrCP^ [8]. Recently, it was found that the pluripotency factor estrogen-related receptor β (ERRβ, Esrrb) contributes to the transcriptional regulation of Dub3 in ESCs [6], however regulation of Dub3 expression during an unchallenged pluripotent cell cycle of ESCs still remains unexplored.

Esrrb is part of the NR3B subgroup that includes three receptors all closely related to estrogen receptors (ERs). A characteristic difference between estrogen receptors (ERs) and ERRs is the constitutive ligand-independent transcriptional activity of ERRs due to the presence of particular amino acids in the putative ligand binding pocket that lock the ligand-binding domain (LBD) in an active conformation [9]. Members of this subgroup are Esrra, Esrrb and the more recently discovered Esrrg [10]. These three related receptors all recognize the consensus DNA sequence TNAAGGTCA (N is any nucleotide), referred to as ERR response element (ERRE), as homo- or hetero-dimers [11], [12]. Among the three members, recent studies have involved Esrrb in regulation of pluripotency in mouse ESCs [13]. Transcriptional activity of ERRs is modulated by coregulator proteins that contain histone acetyltransferase (HAT) activity and rearrange chromatin environment, thus promoting the access of the receptors to their target genes. ERRs display constitutive activity and potentiation by coactivators [10], [14], [15], [16]. Best studied are members of the PGC1 coactivator family that confer “metabolic” activities [17] (and reviewed in [18], [19]) and the p160 family of nuclear coactivators, also know as the steroid receptor coactivator (SRC) family [10], [20]. The latter family consists of three members (NCoA1/NCoA2/NCoA3) and interaction with nuclear receptors occurs through highly conserved LxxLL motifs (called NR boxes) contained in all sequences of the members of the family [21]. NCoA/SRC1A, a splice variant of SRC-1, contains an additional LxxLL motif in its C-terminal part that it is not present in the shorter variant [22], [23].

Recently, it has been shown that Esrrb-dependent activation of key self-renewal genes requires the nuclear receptor coactivator NCoA3 [24]. Depletion of this factor from mESCs, results in downregulation of Esrrb-transcribed genes and loss of pluripotency [24]. Here we report cell cycle-dependent oscillations of Dub3 transcript levels in synchronised, unchallenged mESCs and further increased Dub3 expression upon DNA damage in G1. Unexpectedly, we also observe large cell cycle oscillations of Nanog and the p160 family of coactivators, while mRNA levels of the transcription factors Esrrb and Sox2 display only marginal changes over a full cell cycle. In addition we present evidence that NCoA1 splice variants directly interact with Esrrb and potentiate Dub3 promoter activity. Finally, we report highly specific developmental regulation of all three NCoAs. In summary, we propose that the transcriptional activity of the core transcription factors of the pluripotent network in ESCs is modulated by the relative concentration of a coactivator at different cell cycle phases.

## Materials and Methods

### Plasmids

The firefly luciferase reporter estrogen-responsive pGL3-S2-luc was previously described (ref), and the pGL4.10_Dub3 promoter was constructed by amplifying the 3,2 kb genomic sequence of the proximal promoter of the mouse Dub3 gene [6]. The reporter vector pGL4.10[*luc*] is a Promega product (Cat. number E6651). Importantly, SRC1 and Estrogen related receptor (Esrra, Esrrb and Esrrg) constructs used were previously described and cloned into the same expression vector (pSG5). pSG5-FLAG-mEsrrb, pSG5-FLAG-mEsrrb-ΔCter, pSG5-mEsrra and pSG5-hEsrrg were previously described [11]. The VP16 constructs containing SRC-1 fragments (pSG5-VP16, SRC-1_570–780_, SRC-1_781–988_, SRC-1_989–1240_, SRC-1_1241–1441_, SRC-1_1241–1399_, SRC-1_1241–1388_), pSG5-SRC1A and pSG5-SRC1E. These constructs were kindly provided by O. C. Meijer (LUMC, Leiden, the Netherlands). [22].

### Cell Culture

Monkey CV1 cells and mouse NIH-3t3 cells were routinely cultured and maintained in Dulbecco’s modified Eagle medium (DMEM; GIBCO) supplemented with 10% fetal bovine serum (FBS; GIBCO), L-glutamine, 100 μg/ml penicillin and 100 μg/ml streptomycin. ESCs (CGR8) were cultured on gelatin-coated dishes in the absence of feeder cells with 1,000 U LIF per ml (Millipore). Briefly, ESCs were maintained in Glasgow MEM BHK-21 (GMEM) supplemented with 10% fetal bovine serum, non-essential amino acids, L-glutamine, sodium pyruvate, β-mercaptoethanol. Cells were grown in a humidified atmosphere of 5% CO_2_ at 37°C. All the cell lines were incubated at 37°C and 5% CO_2_.

### Transient Transfection and Reporter Assays

jetPEI^®^ reverse transfection of CV1 cells was performed in 24-wells plates following manufacturer’s protocol. Briefly, DNA was prepared by diluting 900 ng of DNA plasmid (for cotransfection studies for example 450 ng Esrrb and 450 ng SRC1A) plus 50 ng of reporter vector (pGL4.10_Dub3 or pGL4.10). jetPEI solutions were added to the DNA solutions, vortexed and incubated for 30 minutes at room temperature. Cells were counted with Countess Cell Counting Chamber Slides and 0.4% trypan blue (Invitrogen). Per well, 30000 cells were seeded along with jetPEI/DNA mixes. Forty-eight hours post-transfection, cells were harvested and assessed for luciferase activity (Promega). Luciferase data were normalised to pGL4.10 transcriptional activity transfected with the exact same DNA pools. Transient transfection of ESCs was performed using X-tremeGENE 9 DNA according to manufacturer’s protocol (Roche).

### Nocodazole Synchronisation of mESCs

Cells were arrested in prometaphase by nocodazole (Sigma) treatment for 4–8 hours. After mitotic-shake off cells were washed three times in ice-cold PBS and resuspended in full ES growth medium and collected at indicated time for gene expression analysis. For UV-induced DNA damage, 2 hours post release cells were mock- or UV-irradiated (6 J/m^2^) and placed in the incubator at 37°C prior to collection.

### Immunoprecipitation

Following transfection of mESCs with pSG5-FLAG-mEsrrb and pSG5-SRC1A or pSG5-SRC1E using X-treme Gene 9 (Roche), cells were harvested and lysates were incubated with 2 μg anti-FLAG M2 (Sigma; 1 mg/ml; cat. number F1804 ) overnight at 4°C under constant rotation. Protein A-coupled Sepharose beads were extensively washed with lysis buffer and slurry was added to each IP and left 2 h at 4°C under constant rotation. Beads were washed twice with lysis buffer containing protease inhibitors and collected in loading buffer prior PAGE analysis.

### RNA Extraction, cDNA Synthesis and Quantitative Real-time PCR

Total RNA was isolated with TRIzol reagent (Invitrogen). Reverse transcription was carried out using random hexanucleotides (Sigma) and Superscript II First-Strand cDNA synthesis kit (Invitrogen). Quantitative PCR reactions were performed using Lightcycler SYBR Green I Master mix (Roche) on Lightcycler apparatus (Roche). All primers used were intronspanning and to ensure specificity melt-curve analysis were carried out at the end of all PCR reactions. The relative amount of target cDNA was obtained by normalisation using geometric averaging of the following five internal control genes: ACTB, HPRT, HMBS, GAPDH, SDHA. List of all primers used is provided in Supporting Information.

### Neural Conversion of Mouse ESCs

This protocol was as previously described (Ying et al., 2003). ESCs were dissociated and plated in N2B27 medium onto 0.1% gelatine-coated dishes at a density of 1.10^4^ cells/cm^2^. N2B27 medium is a 1∶1 mixture of DMEM/F12 (Gibco) supplemented with modified N2 (25 μg/ml insulin, 100 μg/ml apo-transferrin, 6 ng/ml progesterone (Sigma), 16 μg/ml putrescine (Sigma), 30 nM sodium selenite (Sigma), 50 μg/ml bovine serum albumine (Gibco), Neurobasal medium supplemented with B27 (Gibco), b-mercaptoethanol (0.1 mM) and glutamate (0.2 mM) was also added. The medium was replaced every two days until day 7.

### Satistical Analysis

Graphs showing error bar have been performed at least three times. All data are expressed as mean and error bars indicate the standard deviation. Two-way ANOVA or Student t-test were used to evaluate the differences between groups using Prism software (GraphPad Software). P-value (P) >0.05 was considered as not significant (ns), 0.01<*P*<0.05 as significant and indicated with one asterisks *, 0.001<*P*<0.01 very significant and indicated with two asterisks **, 0.0001<*P*<0.001 extremely significant and indicated with three asterisks *** and *P*<0.0001 extremely significant and indicated with four asterisks ****.

## Results

### DNA Damage-dependent Induction of Dub3 Expression in mESCs

**Figure 1:**
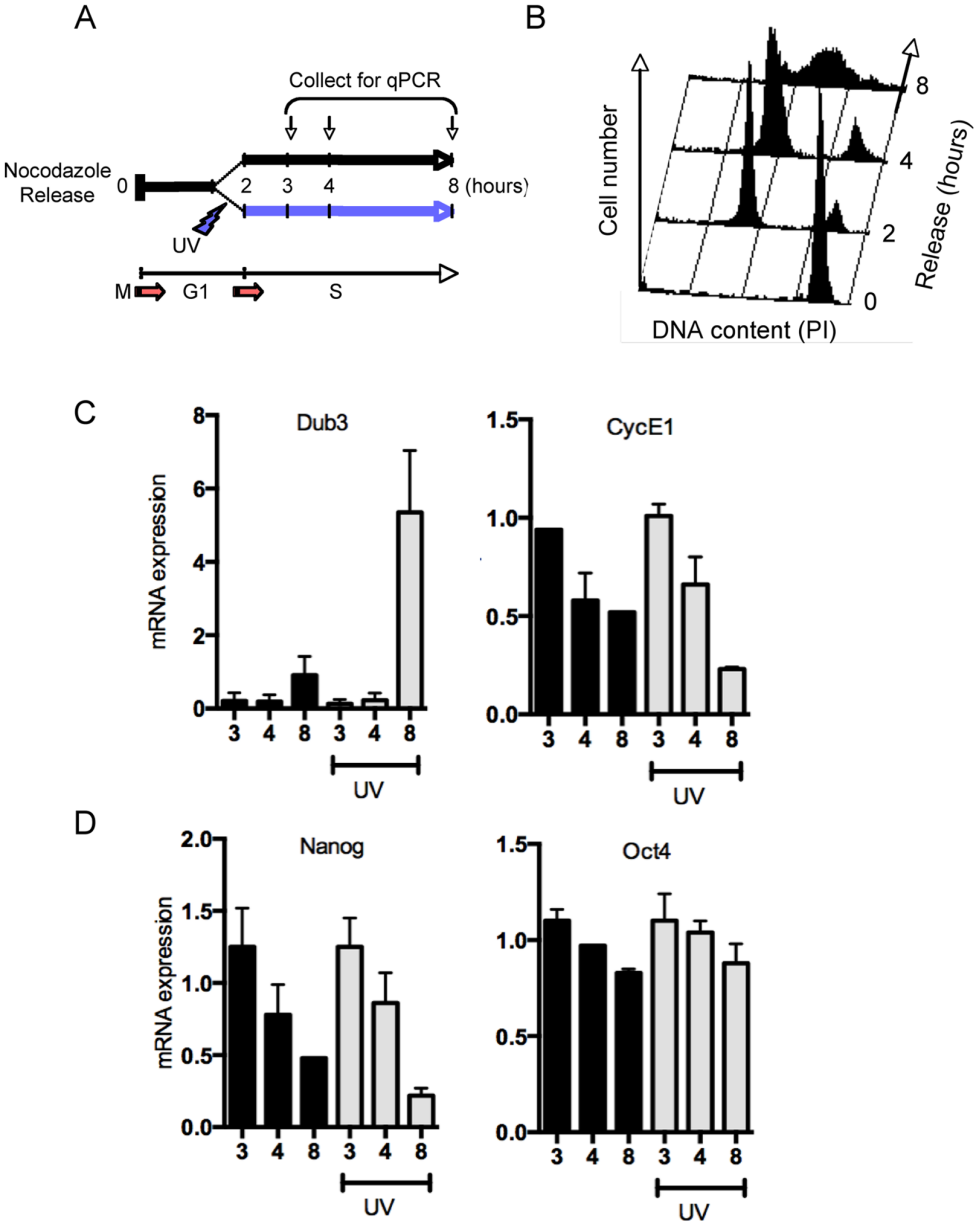
Increase of Dub3 expression upon UV-induced DNA damage in mESCs. (A) Schematic representation of the experimental setup. Mouse ESCs were nocodazole arrested and 2 hours post released mock or UV-irradiated (6 J/m^2^) and collected at indicated time points for RNA extraction and gene expression analysis. (B) FACS analysis of total DNA content (PI staining) of mES cells released from nocodazole collected at indicated time points (hours after release). (C) Mouse ESCs cells released from nocodazole and collected at indicated time points (hours after release) for qPCR quantification of Dub3 and Cyclin E1 mRNA levels. Data was normalized to multiple reference genes and expressed as average of three biological replicates. Error bars indicate standard deviation. (D) qPCR quantification of Nanog and Oct4 mRNA normalised to multiple reference genes from mESCs released from nocodazole and collected at indicated time points (hours after release). expressed as average of three biological replicates. Error bars indicate standard deviation. (See also Figure S1).

Dub3 is a deubiquitylase that stabilizes Cdc25A protein levels by counteracting Cdc25A polyubiquitylation, mediated by both APC^Cdh1^ and SFC^β-TRCP^ E3 ubiquitin ligases [6], [7]. Since the regulation of Dub3 expression during the cell cycle of ESCs is not known, we studied Dub3 gene expression during the cell cycle in the absence or presence of DNA damage. To this end, we synchronised ESCs by nocodazole treatment prior to mock or UV-irradiation and analyzed gene expression during the following 6 hours post irradiation (Figure 1A and 1B). Interestingly, we observed that Dub3 transcript levels display a marked cell cycle-dependent oscillation, significantly increasing during S-phase and strongly upregulated upon UV-irradiation (Figure 1C, left panel). As expected, Cyclin E1 levels were high upon nocodazole release and promptly decreased upon S-phase entry, which occurs 2–3 hours after release (Figure 1C, right panel) and as previously reported [6]. Surprisingly, we also observed cell cycle-dependent oscillations of Nanog, very similar to Cyclin E1 (Figure 1D, left panel), whereas Oct4 transcript levels did not display significant variations (Figure 1D, right panel). Importantly, UV damage did not stimulate Nanog expression, but rather decreased it, as opposed to Dub3 expression, which was markedly increased. In line with previous experiments [25], UV-induced DNA damage led to increased Noxa mRNA levels, likely through a p53-mediated response, whereas Chk1 levels showed neither UV, nor cell cycle-dependent changes (Figure S1A). Interestingly, and in contrast to Noxa expression, the UV-induced gene expression of Dub3 is not p53-dependent, since it is equally observed in p53−/− mESCs (Figure S1B), suggesting regulation by another transcriptional pathway (see next paragraph).

Since in mESCs Dub3 contributes to relaxation of the G1/S checkpoint by constitutive stabilization of Cdc25A even upon DNA damage [6], these data suggest that Dub3 increase could be beneficial for checkpoint recovery by further contributing to Cdc25A stabilisation. In line with this model, we observed that ectopic expression of Dub3 in mouse embryonic fibroblasts (MEFs) NIH-3t3 cells, that spend most of their time in G1 (Figure S1C), not only stabilized Cdc25A levels upon UV-induced DNA damage, confirming a previous report [7], but also prevented Cdk2 phosphorylation caused by prompt proteasomal degradation of the Cdc25A phosphatase (Figure S1D, compare lane 2 and 4). This observation confirms that increased Dub3 abundance stabilizes Cdc25A and blunts the DNA damage checkpoint response.

### Cell Cycle-dependent Oscillations of Dub3 and NCoAs in mESCs

**Figure 2:**
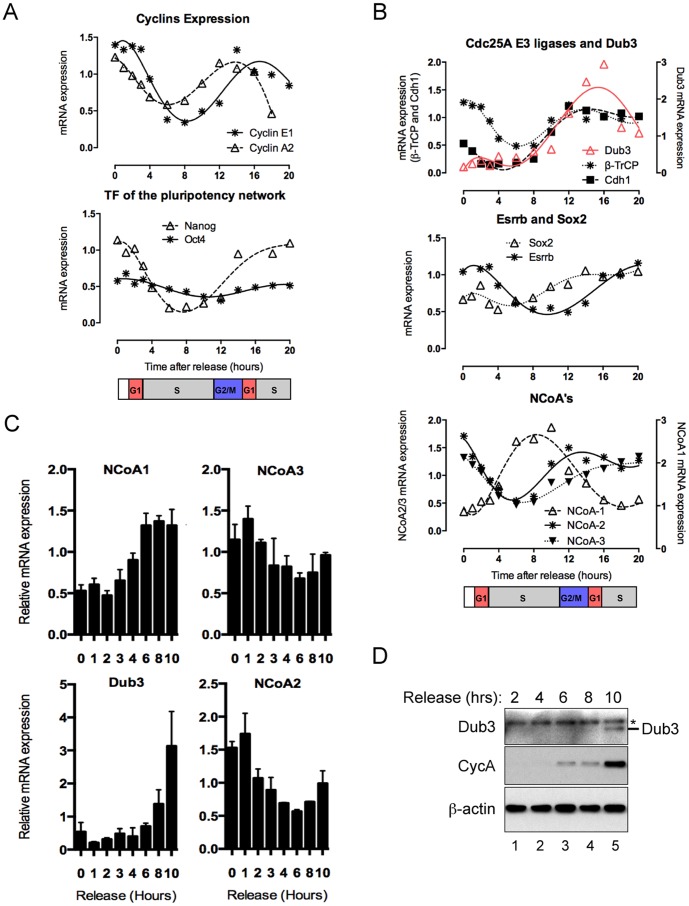
Cell cycle dependent oscillations of Nanog, Dub3 and NCoAs. (A) qPCR quantification of Cyclin E1, Cyclin A2, Nanog and Oct4 mRNA normalised to multiple reference genes from mESCs released from nocodazole collected during a full cell cycle at indicated time points (hours after release) (B) qPCR quantification of β-TrCP, Cdh1 and Dub3 mRNA (upper panel), NCoA1, NCoA2 and NCoA3 mRNA (middle panel) and Esrrb and Sox2 mRNA (lower panel) normalised to multiple reference genes from mESCs released from nocodazole collected during a full cell cycle at indicated time points (hours after release). (C) qPCR quantification of NCoA1, NCoA2, NCoA3 and Dub3 mRNA normalised to multiple reference genes from mESCs released from nocodazole collected at indicated time points (hours after release). Data is shown as average of three biological replicates and the error bars indicate the standard deviation. (D) Western blot analysis of mESCs released from nocodazole and harvested at indicated time points (hours after release). Antibodies used for immunoblotting of proteins are indicated and * indicates non-specific band. (See also Figure S2).

To further analyse the cell cycle-dependent variations of Dub3 transcript levels, we released mESCs from a nocodazole block (as shown in Figure 1A and 1B) and collected total RNA samples over a longer time course (20 hours) for gene expression analysis. As previously reported in somatic cells [26], distinct oscillations of Cyclin E1 and A2 were observed at their respective cell cycle stages (Figure 2A, upper panel). Surprisingly, and consistent with data shown on Figure 1, we also observed strong fluctuations in Nanog transcript levels, being high at the G1/S border, while Oct4 mRNA levels displayed only marginal differences (Figure 2A, lower panel). Interestingly, while β-TrCP and Cdh1 transcript levels both paralleled the expected cell cycle dependent changes in activity of APC/C [27], being high in G1 and G2/M phase, the amplitude of Dub3 oscillations were found to be much higher and to reach a maximum in G1, prior to S phase, as monitored by Cyclin E1 levels (Figure 2B, upper panel). Of note, upon nocodazole release, Dub3 transcript level was very low suggesting that cell cycle-associated gene expression of Dub3 occurs prior to prolonged arrest in prometaphase. Indeed, increase in mRNA was detected 8–10 hours after release (Figure 2B). Since Dub3 proximal promoter contains three consensus Esrrb and two Sox2 binding sites [6], we analyzed expression of both transcription factors that belong to the key pluripotent transcription factors network in mESCs. Although mRNA of Sox2 oscillated during the cell cycle, the amplitude was somehow limited as compared to that observed for Nanog and Dub3 (Figure 2B, middle panel). In contrast Esrrb showed a lower degree of oscillation being most abundant at G1/S transition.

We also analyzed the expression of nuclear coactivators NCoA1-3 since Esrrb function in mESCs has recently been reported to be tightly coupled to NCoA3 [24]. Strikingly, NCoA1 expression was highly increased during S-phase prior to Dub3 expression, at a time when NCoA2 and NCoA3 expression were low (Figure 2B, lower panel). The above-mentioned observations were corroborated by analysis of multiple independent experiments over a shorter time frame (Figure 2C). NCoA1 expression increased 4–6 hours after release, when most cells have entered S-phase [6], and prior to the increase of Dub3 mRNA levels. In contrast, NCoA2 and NCoA3 inversely correlated with Dub3 expression, suggesting that NCoA1 may contribute to the strong cell cycle-dependent oscillation of Dub3 expression levels. In support to this possibility, expression of the individual NCoA1 splice variants (NCoA/SRC1A or NCoA/SRC1E) in mESCs, both led to an increase of endogenous Dub3 mRNA without affecting Esrrb gene expression (Figure S2). Finally, we found that protein abundance of Dub3 during the cell cycle finely matched transcript levels, confirming previous results showing that protein expression of Dub3 in mouse ES cells strictly correlates with mRNA expression in both asynchronously growing ES cells and during neural conversion [6]. Interestingly, Dub3 protein levels during the cell cycle clearly showed similar expression profiles as cyclin A, which is induced at the end of S-phase (Figure 2D).

### Functional Interaction of NCoA1 Splice Variants with Esrrb on Dub3 Promoter

**Figure 3:**
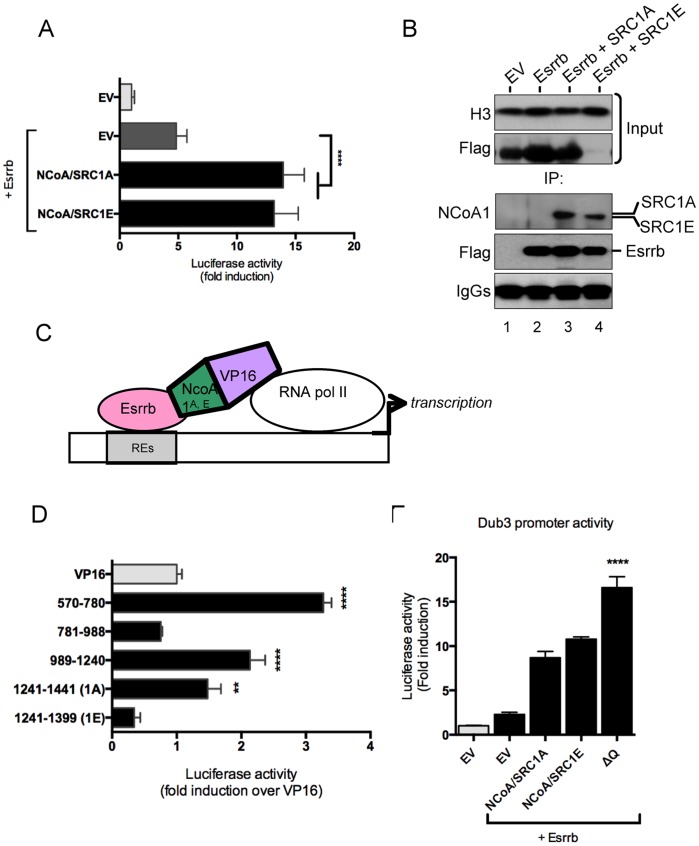
Direct interaction between NCoA1 splice variants and Esrrb. (A) CV1 cells were transfected with equal amount of plasmid DNA (50 ng reporter/450 ng Esrrb/450 ng NCoA) and luciferase activity was measured 48 hours after transfection. Data were normalised to pGL4.10 empty vector. Data is shown as average of fold induction of six biological replicates and the error bars indicate the standard deviation. Four asterisks indicate that *P*<0.0001 extremely significant. (B) NCoA1 splice variants coimmunoprecipitate with Esrrb. Mouse ESCs were transfected using Xtreme gene (Roche) with equal amounts of Esrrb and NCoA. Cells were harvested 48 hours post transfection, and Esrrb was immunoprecipitated. IP’s were analysed by western blotting. Histone H3 was used as an input control. (C) Mammalian one-hybrid assay. Schematic representation displaying the rational of the experiment. REs indicate Esrrb Responsive Elements within the DuB3 promoter. (D) CV1 cells were transfected with equal amount of plasmid DNA (pGl4.10_Dub3, VP16 constructs and pSG5-Esrrb) and luciferase activity was measured 48 hours after transfection. Data is shown as average of fold induction of six biological replicates and the error bars indicate the standard deviation. Two asterisks indicate that 0.001<*P*<0.01 is very significant and four asterisks indicate that *P*<0.0001 extremely significant. (E) CV1 cells were transfected with equal amount of plasmid DNA and luciferase activity was measured 48 hours after transfection. Data is shown as average of fold induction of six biological replicates and the error bars indicate the standard deviation. Four asterisks indicate that *P*<0.0001 extremely significant. (See also Figure S3).

**Figure 4:**
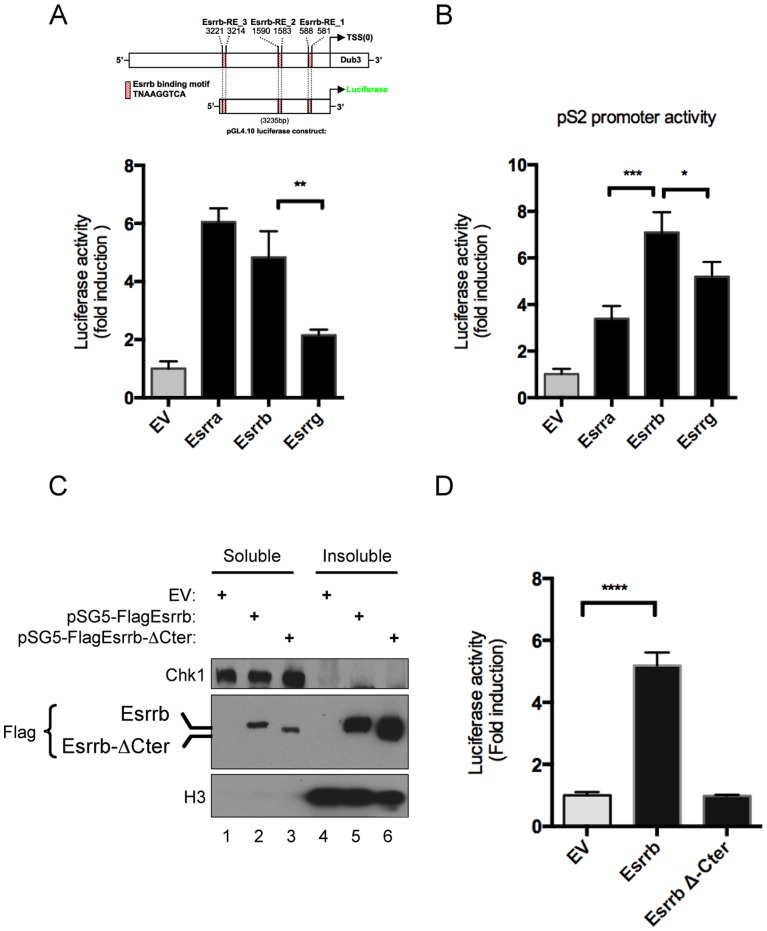
Transcriptional regulation of Dub3 by Esrrb is dependent on coactivator recruitment. (A) Schematic representation of the proximal promoter of the mouse Dub3 gene and reporter construct is shown. Transcriptional activity of the ERRs family of orphan receptors on the Dub3 promoter. CV1 cells were transfected with equal amount of plasmid DNA and luciferase activity was measured 48 hours after transfection. Data is shown as average of fold induction of six biological replicates and the error bars indicate the standard deviation. Two asterisks indicate that 0.001<*P*<0.01 is very significant. (B) Luciferase reporter assay of transfected CV1 cells. Luciferase activity was measured 48 hours after jetPEI DNA reverse co-transfection in 24-well plates with reporter plasmid for pS2. All three isoforms of the ERR family of orphan receptors were individually tested for their activity on both pS2 and Dub3 promoter sequences. Grey bars indicate control transfection with empty vector. Data is shown as average of fold induction of six biological replicates and the error bars indicate the standard deviation. (C) Western blot analysis CV1 cells after transfection with equal amount of pSG5-Esrrb and pSG5-Esrrb-ΔCter. Cells were harvested and fractionated in soluble and insoluble (chromatin- fractions. Chk1 and Histone H3 were used as fractionation controls. (D) Transcriptional activity of Esrrb on the Dub3 promoter is AF2 dependent. CV1 cells were transfected with equal amount of plasmid DNA and luciferase activity was measured 48 hours after transfection. Data is shown as average of fold induction of six biological replicates and the error bars indicate the standard deviation. Four asterisks indicate that *P*<0.0001 extremely significant.

To address the specific role of the two splice variants NCoA/SRC1A and NCoA/SRC1E on Esrrb-mediated Dub3 transcription, we individually cotransfected each coactivators with Esrrb and measured the activity of a luciferase reporter gene. To limit the contribution of endogenous expressed receptors in the outcome of the experiment, we performed the transcription assay in CV1 cells that have very low levels of endogenous steroid receptor. We observed that expression of both splice variants resulted in highly comparable stimulation of Esrrb-mediated transcriptional activity (Figure 3A). Next, to determine if Esrrb and NCoA1 could co-immunoprecipitate, we cotransfected Esrrb along with NCoA1 splice variants in mESCs. The antibody used for NCoA1 detection (Figure S3A) was raised against a common part to both splice variants of human origin (amino acids 350–690), and therefore recognizes both SRC1 variants. This antibody detected both SRC1A and SRC1E in Esrrb immunoprecipitates in mESCs extracts, suggesting that these proteins aggregate in common complexes (Figure 3B). Of note, the slight difference in electrophoretic mobility observed on SDS-PAGE between the two variants reflects the presence of a smaller C-terminal sequence of the NCoA/SRC1E isoform (Figure 3B). Recently the interaction of Esrrb with the transcription factor Dax1 was reported to depend on LxxLL motifs [28] that are also contained in both the NCoA/SRC1A and NCoA/SRC1E protein sequences. Therefore, to assess whether this motif is also involved in interaction of NCoA1 with Esrrb we used a mammalian one-hybrid system composed of SRC1 fragments fused to the strong VP16 activation domain (Figure 3C and Figure S3B for a schematic representation of the mutants). NCoA/SRC1A protein sequence encloses one additional LxxLL motif in its splice specific C-terminal domain that it is not present in the shorter variant of NCoA/SRC1E. As anticipated, the nuclear receptor interacting domain that contains three LxxLL motifs (570–780) showed increased transcriptional activity on the Dub3 promoter in presence of Esrrb compared to the VP16 control, indicative of recruitment of the VP16 activator domain (AD) in vicinity of the promoter (Figure 3D). In line with the expectation that the LxxLL motifs mediate this interaction, the NCoA/SRC1A sequence that contains the additional splice variant-specific LxxLL motif, also displayed increased Esrrb-mediated Dub3 transcriptional activity. In contrast, the NCoA/SRC1E-specific fragment that does not contain a LxxLL motif did not increase Esrrb-mediated transcription (Figure 3D). Unexpectedly, expression of the Q-rich containing domain fused to VP16 AD (989–1240) also resulted in an increase of Esrrb-mediated transcription suggesting interaction with Esrrb (Figure 4D). Curiously, deletion of the Q-rich domain of the NCoA/SRC1E sequence resulted in further potentiation of Esrrb-mediated Dub3 transcriptional activity (Figure 4E). Altogether these data provide evidence of functional and possibly protein interaction between NCoA1 and Esrrb that results in potentiation of Esrrb-mediated transcription on the Dub3 promoter.

### Transcriptional Regulation of Dub3 by the ERR Family of Receptors

The proximal promoter of the mouse Dub3 promoter contains three consensus Esrrb binding sites (TNAAGGTCA; Figure 4A, schematic representation). Since the three members of the ERR family of orphan nuclear receptors Essra, Esrrb and Esrrg all recognize the same consensus sequence, we tested the contribution of each individual ERR on transcriptional activity of the Dub3 proximal promoter compared to the pS2 promoter, a widely used breast cancer marker gene shown to be regulated by ERRs, used here as a positive control (Figure 4A and 4B). Interestingly, upon transfection of equal amounts of vectors expressing each of the ERRs in CV1 cells, Esrra and Esrrb strongly induced transcription on Dub3 promoter in a luciferase reporter assay, while Esrrg-driven transcription activity was much weaker. Importantly, and in line with previous reports [16], [29] all ERRs stimulated transcriptional activity on the pS2 proximal promoter although to various extents (Figure 4B). Altogether, these data support the idea that transcriptional regulation is not restricted to the sequence of the response element itself, but underline the significance of adjacent sequences that likely convey additional information. Finally, to control specificity, we transfected equal amounts of wild-type Esrrb (pSG5-Esrrb) or a dominant negative mutant (pSG5-Esrrb-ΔCter) lacking the AF-2 portion of the ligand binding domain (LBD) essential for coactivator recruitment. In line with their function as transcription factors, we observed enrichment of both Esrrb and Esrrb-ΔCter in the insoluble chromatin fraction (Figure 4C). As expected, only wild type Esrrb and not the C-terminally truncated receptor increased Dub3 transcriptional activity (Figure 4D), indicating that coactivator recruitment through the C-terminus that contains the AF2 domain is essential to the Esrrb-mediated transcriptional response on the Dub3 promoter.

### Developmental Control of NCoA Gene Expression

**Figure 5:**
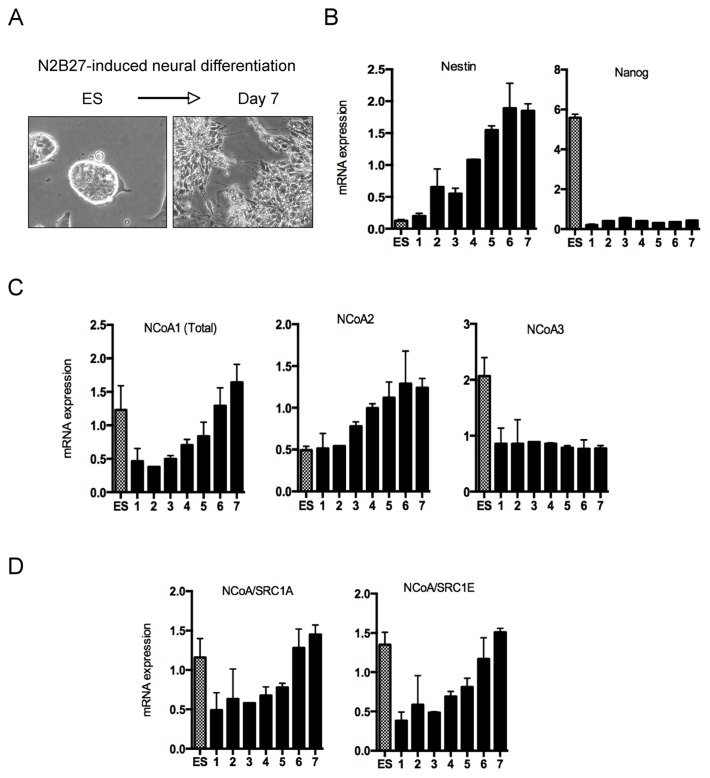
Developmental regulation of NCoA’s gene expression. (A) Representative images of an ES cell colony and resulting neural stem cells after plating during 7 days in N2B27 medium (clearly visible are the rosette formation indicative of neural stem cell differentiation). (B) qPCR quantification of Nestin (cell fate marker) and Nanog (pluripotency marker) mRNA expression during neural conversion. Data is shown as average of three biological replicates and the error bars indicate the standard deviation. (C) qPCR quantification of all three members of the NCoA family mRNA expression during neural conversion. Data is shown as average of three biological replicates and the error bars indicate the standard deviation. (D) qPCR quantification of NCoA1 specific splice variants SRC1A and SRC1E expression during neural conversion. Primers were designed to specifically determine transcript levels of both splice variants. Data is shown as average of three biological replicates and the error bars indicate the standard deviation.

Esrrb and Dub3 levels are high in pluripotent ESCs and rapidly drop upon differentiation [6]. Since NCoA1 interacts and regulates Esrrb transcriptional activities in mESCs, we analyzed the expression profile of the p160 family of nuclear coactivators during differentiation. ESCs were homogenously differentiated toward neuroectoderm lineage by plating them in N2B27 medium (Figure 5A). Loss of pluripotency and acquisition of neural identity was monitored by gene expression of Nanog and Nestin respectively (Figure 5B). Interestingly, none of the three members of NcoAs showed overlapping developmental regulation of gene expression (Figure 5C). While NCoA1 levels rapidly dropped by more than 60% at onset of differentiation (day 1), mRNA levels rose again after day 4 (Figure 5C, left panel), suggesting additional role of NCoA1, since Esrrb transcript levels are hardly detectable after day 2 [6]. Of the three members, NCoA2 levels steadily increased over the whole differentiation period (Figure 5C, middle panel). Finally, and in line with previous work [24], we observed a drop of NCoA3 levels by 50% during the first 24 hours that remained unchanged afterwards (Figure 5C, right panel). Interestingly, both NCoA/SRC1A and NCoA/SRC1E specific transcripts showed highly comparable expression profiles, indicating that despite changes in gene expression upon differentiation, the activity of the splicing machinery on these variants does not vary (Figure 5D). In summary, these data show strong developmental regulation of NCoA transcript levels, suggesting individual impacts of all three members on nuclear receptor-dependent transcription eventually contributing thereby to the differentiation program and cell fate decisions.

## Discussion

In this study, we surprisingly observed that Dub3, Nanog, and the p160 family of coactivators transcript levels display strong cell cycle oscillations in synchronised mESCs. Interestingly, we observed that Dub3 expression is further stimulated upon UV-damage in G1. Hence, although Dub3 is abundant in mESCs, a further increase in expression occurs after UV damage, suggesting a role of Dub3 during S-phase, possibly in checkpoint recovery by facilitating G2 to M–phase transition by dephosphorylation of CDK1 [8]. Expression of Dub3 is also observed in cells in which p53 expression has been abrogated, suggesting an alternative DNA damage induced transcriptional control that may implicate the nuclear family of estrogen receptors (ERRs) and their co-activators (see below). In this respect, previous work has shown that the expression of the DNA damage-inducible CDK inhibitors p21 and p27 is also under control of ERRs [30], [31], [32].

Nanog, Oct4, Sox2 and Esrrb are core components of the pluripotency network of mESCs. Nanog is a transcription factor that functions in maintaining self-renewal and its overexpression confers pluripotency [33]. In line with our data in mouse ESCs, a very recent report has shown that Oct4 and Sox2 do not display a consistent pattern of periodicity in the cell cycle of human ESCs [34]. However, we observed marked oscillations of both Nanog and Cyclin E1 transcript levels in mESCs during cell cycle progression, reaching maximal levels prior to G1/S transition, suggesting a role of Nanog in cell cycle-associated events, which were not observed in hESCs [34]. Although it cannot be excluded that these molecular pathways are divergent between human and mouse ESCs, the function of Nanog to accelerate S-phase entry could be conserved since, a role for Nanog in G1 to S transition in human embryonic stem cells has already been documented [35]. Also, we cannot rule out differences in the technical approach used in our study compared to that of Singh and colleagues in human ESCs [34], but it might be that the regulation of both genes is species-specific. Indeed, a growing body of evidence suggests that specific components of the mechanisms controlling early developmental stages could differ between mouse and human [36], [37], although expression of the putative human Dub3 ortholog USP17L2 was shown to be also cell cycle-regulated and required for G1 to S-phase transition [38].

Intriguingly, we also observed strong oscillations of the transcript levels for the p160 family of coactivators NCoA1-3. Importantly, while NCoA1 expression preceded Dub3 increase during the cell cycle, NCoA2 and NCoA3 displayed an inverse correlation, suggesting a role of NCoA1 in Dub3 expression. Analysis of Dub3 promoter activity in a reporter assay confirmed an involvement of both NCoA1 splice variants in strongly potentiating Esrrb-mediated transcription on the Dub3 promoter. By means of a mammalian one-hybrid assay and immunoprecipitation experiments, we have also provided evidence for a direct interaction between Esrrb and NCoA1. Finally, mESCs neural conversion showed that developmental regulation of gene expression is specific to each coactivator. Altogether, we propose that the relative concentration of coactivators at a given stage of the cell cycle might be sufficient to dictate the transactivation potential of transcription factors such as Esrrb and Nanog that are core components of the self-renewal machinery of mESCs. These findings further highlight the complexity of the regulatory network of transcription factors in pluripotent mESCs and may explain why downregulation of Esrrb does not completely abolish Dub3 gene expression in mESCs [6].

In line with a recent report showing that interaction of Dax1 with Esrrb is mediated through LxxLL motifs within the Dax1 protein sequence [28], we found that truncated NCoA1 splice variants that contain LxxLL could functionally interact with Esrrb. Although the interaction of DAX1 and NCoA1 is possibly conferred by similar binding motifs, their role on transcription is opposite. DAX1 is an orphan nuclear receptor and is known to function as a transcriptional repressor whereas NCoA1 stimulates transcription through its histone acetyltransferase activity (HAT) and recruitment of additional factors such as CBP/p300 or the methyltransferase CARM-1 [39], [40], [41]. Because both proteins contain LxxLL motifs, competition for binding the receptor is anticipated. Surprisingly, we also found an interaction through the Q-rich domain, which seemed to convey inhibitory signals since deletion of this region resulted in increased Esrrb-mediated Dub3 transcription (Figure 3). In light of the role of NCoA3 in sustaining embryonic stem cell self-renewal and reprogramming [24] and given our data, it seems very likely that in a manner similar to NCoA3, NCoA1 splice variants are critical determinants of ESCs biology. More experiments will be required to support this possibility. The different periodicity of expression of NCoA1 and the other two members of the family further suggests that these coactivators have different functions.

In summary, in this work we have presented evidence that NCoAs and Nanog expression in mouse ESCs displays strong cell cycle dependent oscillations, and propose that the transcriptional activity of the core transcription factors of the pluripotent network in ESCs may very well be modulated by the relative concentration of a coactivator given at different cell cycle phases. Since reprogramming of somatic cells into induced pluripotent stem cells (iPSs) is achieved by constitutive expression of pluripotency factors that do not mimic the natural cell cycle of ESCs, understanding this regulation may uncover new molecular targets to optimise generation of iPS cells with limited theratogenic potential.

## Supporting Information

Figure S1 (A) qPCR quantification of Noxa (control for UV treatment), and Chk1 mRNA normalised to multiple reference genes from mESCs released from nocodazole collected at indicated time points (hours after release). (B) Wild type (wt) and p53−/− mouse ESCs were UV-irradiated (10 J/m^2^) and collected at indicated time points for qPCR quantification of p53, Noxa and Dub3 mRNA levels. Data were normalised to multiple reference genes and expressed as average of multiple biological replicates. Error bars indicate standard deviation. (C) FACS analysis of asynchronyously growing NIH-3t3 cells. Cells were pulse labelled with BrdU 30 minutes prior sampling and analysed by flow cytometry. (D) NIH-3t3 cells were transfected with empty vector (EV) or pcDNA-HADub3 and mock or UV-irradiated (20 J/m^2^) 48 hours post transfections. Cells were collected 30 minutes after treatment and processed for western blot analysis. (See also Figure 1).
(TIF)

Figure S2 Mouse ESCs were transfected with equal amount of NCoAs and collected 48 hours post transfection for qPCR quantification of Esrrb and Dub3 mRNA normalised to multiple reference genes. Data is shown as average of multiple biological replicates and the error bars indicate the standard deviation. (See also Figure 2).
(TIF)

Figure S3 (A) Testing of the SRC1 antibody. Western blot analysis of mESCs transfected with NCoA/SRC1A isoform DNA plasmid. Detection of the Histone H3 is used as loading control. (B) Schematic representation of SRC1 splice variants and VP16 chimeras used for protein-protein interaction assays. The purple bars indicate LxxLL motifs. NR (nuclear receptor box), AD1 (Activation domain 1), AD2 (Activation domain 2) and Q stands for glutamine rich domain.
(TIF)

Text S1 Supporting materials and methods.
(DOC)
