# Correction: Cell Cycle-Dependent Expression of Dub3, Nanog and the p160 Family of Nuclear Receptor Coactivators (NCoAs) in Mouse Embryonic Stem Cells

**DOI:** 10.1371/journal.pone.0105649

**Published:** 2014-08-08

**Authors:** The PLOS ONE Staff

Figure 3B is incorrect. The authors have provided a corrected version here.

**Figure 3:**
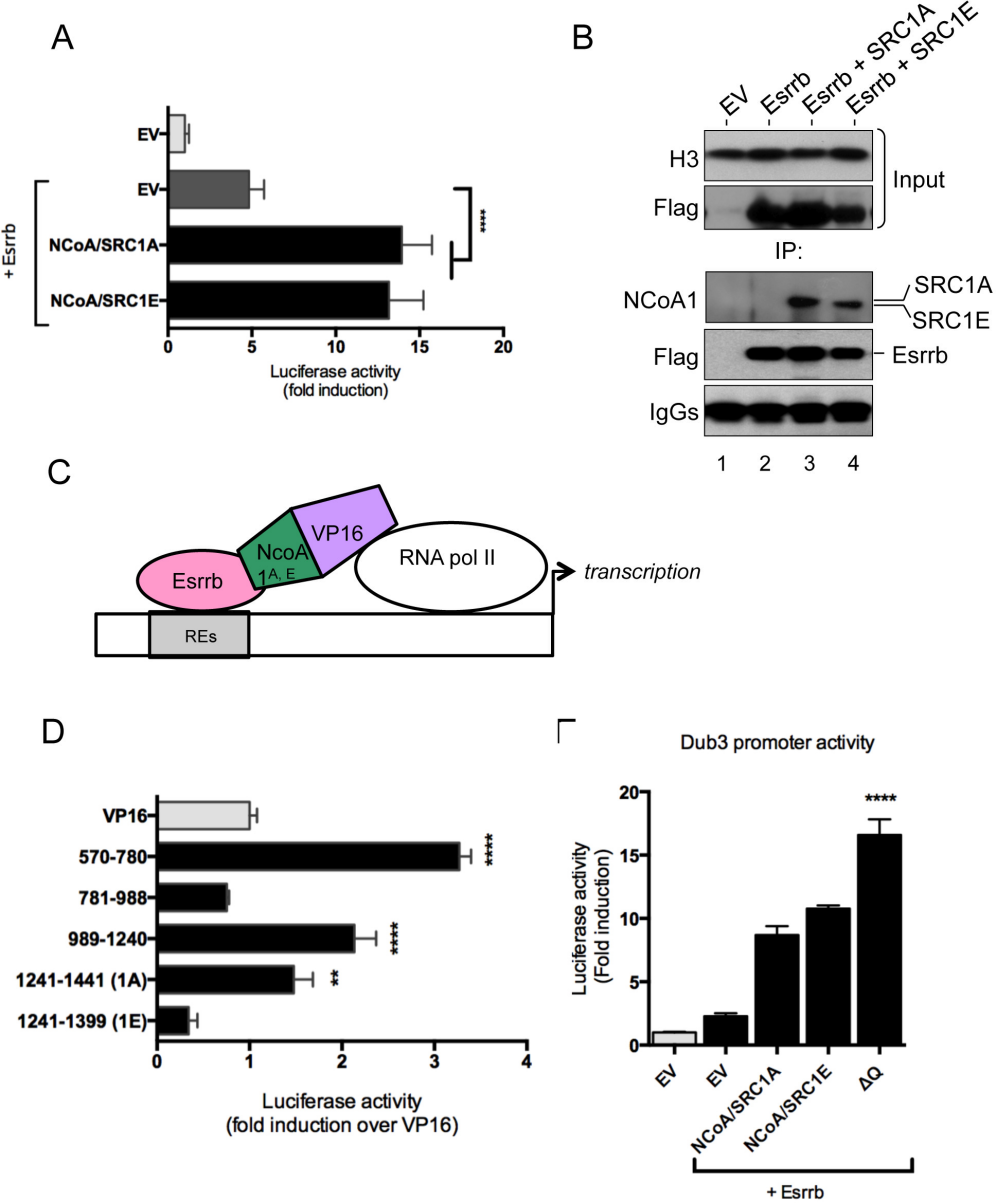
Direct interaction between NCoA1 splice variants and Esrrb. (A) CV1 cells were transfected with equal amount of plasmid DNA (50 ng reporter/450 ng Esrrb/450 ng NCoA) and luciferase activity was measured 48 hours after transfection. Data were normalised to pGL4.10 empty vector. Data is shown as average of fold induction of six biological replicates and the error bars indicate the standard deviation. Four asterisks indicate that P<0.0001 extremely significant. (B) NCoA1 splice variants coimmunoprecipitate with Esrrb. Mouse ESCs were transfected using Xtreme gene (Roche) with equal amounts of Esrrb and NCoA. Cells were harvested 48 hours post transfection, and Esrrb was immunoprecipitated. IP’s were analysed by western blotting. Histone H3 was used as an input control. (C) Mammalian one-hybrid assay. Schematic representation displaying the rational of the experiment. REs indicate Esrrb Responsive Elements within the DuB3 promoter. (D) CV1 cells were transfected with equal amount of plasmid DNA (pGl4.10_Dub3, VP16 constructs and pSG5-Esrrb) and luciferase activity was measured 48 hours after transfection. Data is shown as average of fold induction of six biological replicates and the error bars indicate the standard deviation. Two asterisks indicate that 0.001<P<0.01 is very significant and four asterisks indicate that P<0.0001 extremely significant. (E) CV1 cells were transfected with equal amount of plasmid DNA and luciferase activity was measured 48 hours after transfection. Data is shown as average of fold induction of six biological replicates and the error bars indicate the standard deviation. Four asterisks indicate that P<0.0001 extremely significant. (See also Figure S3).
