# Supplementary figures and images for: Cell Cycle-Dependent Expression of Dub3, Nanog and the p160 Family of Nuclear Receptor Coactivators (NCoAs) in Mouse Embryonic Stem Cells
Source: PLoS One. 2014 Apr 2;9(4):e93663. doi: 10.1371/journal.pone.0093663 (PMC3973558; doi:10.1371/journal.pone.0093663)

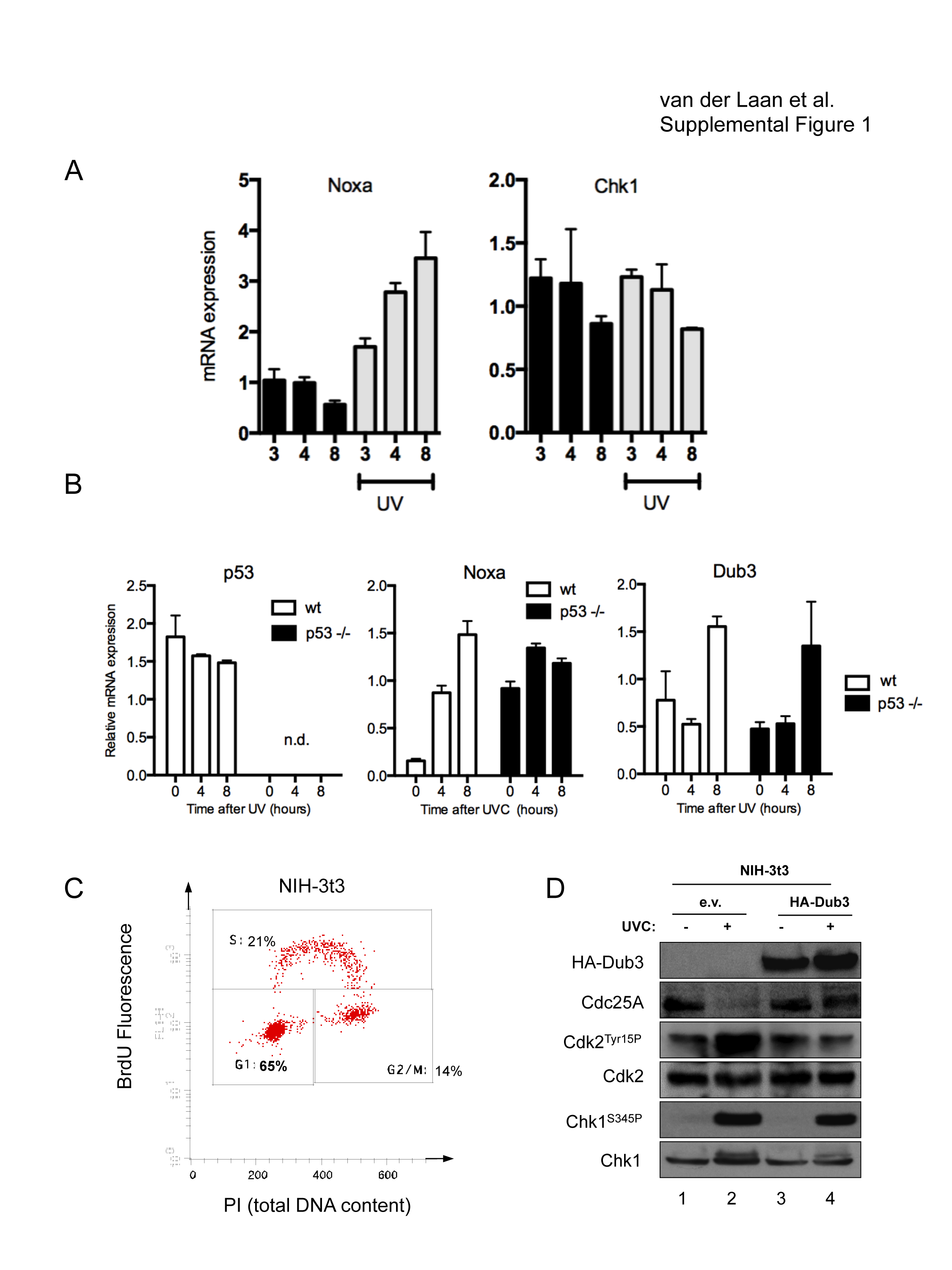

Supplement: Figure S1 — (A) qPCR quantification of Noxa (control for UV treatment), and Chk1 mRNA normalised to multiple reference genes from mESCs released from nocodazole collected at indicated time points (hours after release). (B) Wild type (wt) and p53−/− mouse ESCs were UV-irradiated (10 J/m2) and collected at indicated time points for qPCR quantification of p53, Noxa and Dub3 mRNA levels. Data were normalised to multiple reference genes and expressed as average of multiple biological replicates. Error bars indicate standard deviation. (C) FACS analysis of asynchronyously growing NIH-3t3 cells. Cells were pulse labelled with BrdU 30 minutes prior sampling and analysed by flow cytometry. (D) NIH-3t3 cells were transfected with empty vector (EV) or pcDNA-HADub3 and mock or UV-irradiated (20 J/m2) 48 hours post transfections. Cells were collected 30 minutes after treatment and processed for western blot analysis. (See also Figure 1). (TIF) [file pone.0093663.s001.tif]

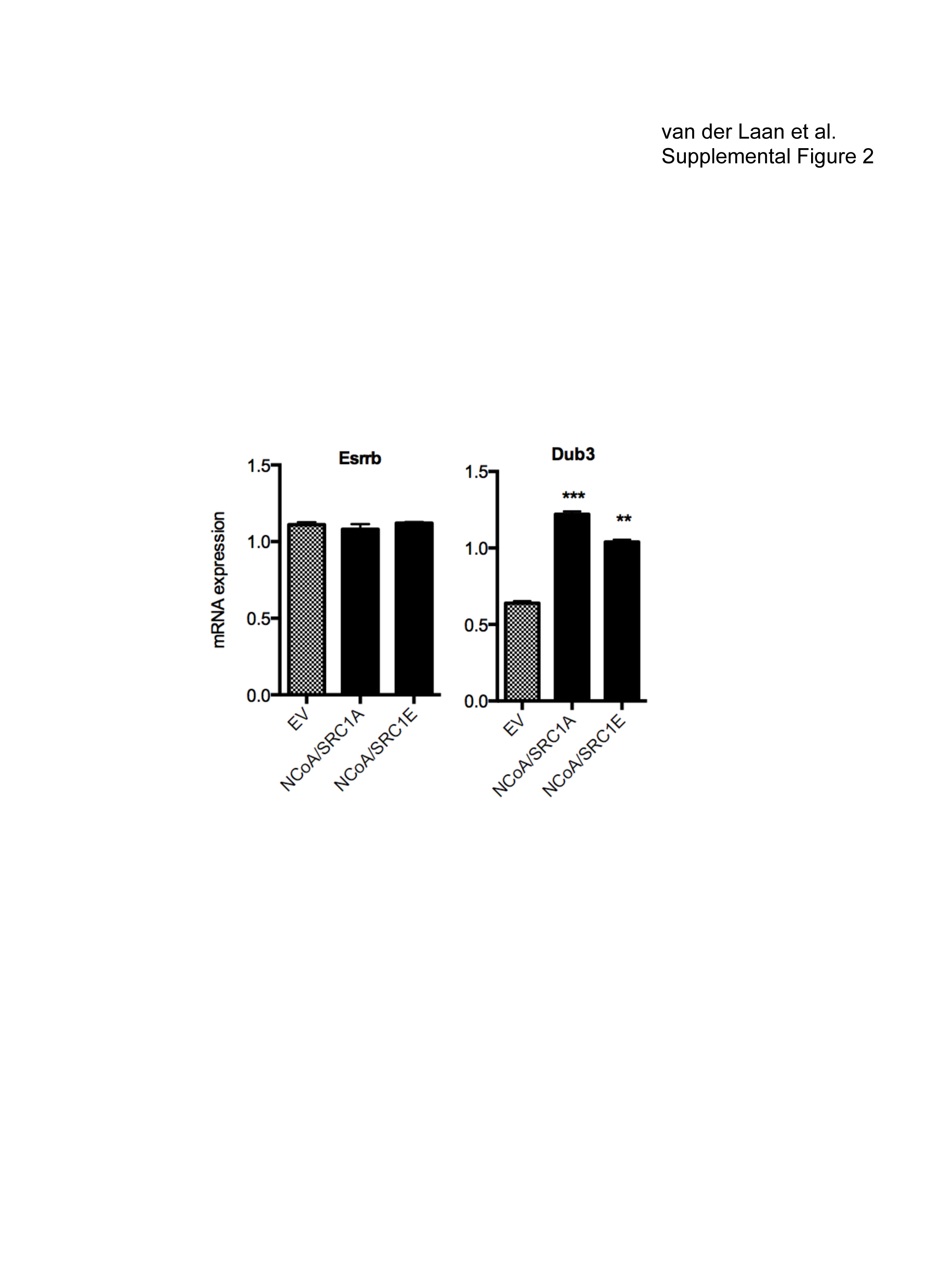

Supplement: Figure S2 — Mouse ESCs were transfected with equal amount of NCoAs and collected 48 hours post transfection for qPCR quantification of Esrrb and Dub3 mRNA normalised to multiple reference genes. Data is shown as average of multiple biological replicates and the error bars indicate the standard deviation. (See also Figure 2). (TIF) [file pone.0093663.s002.tif]

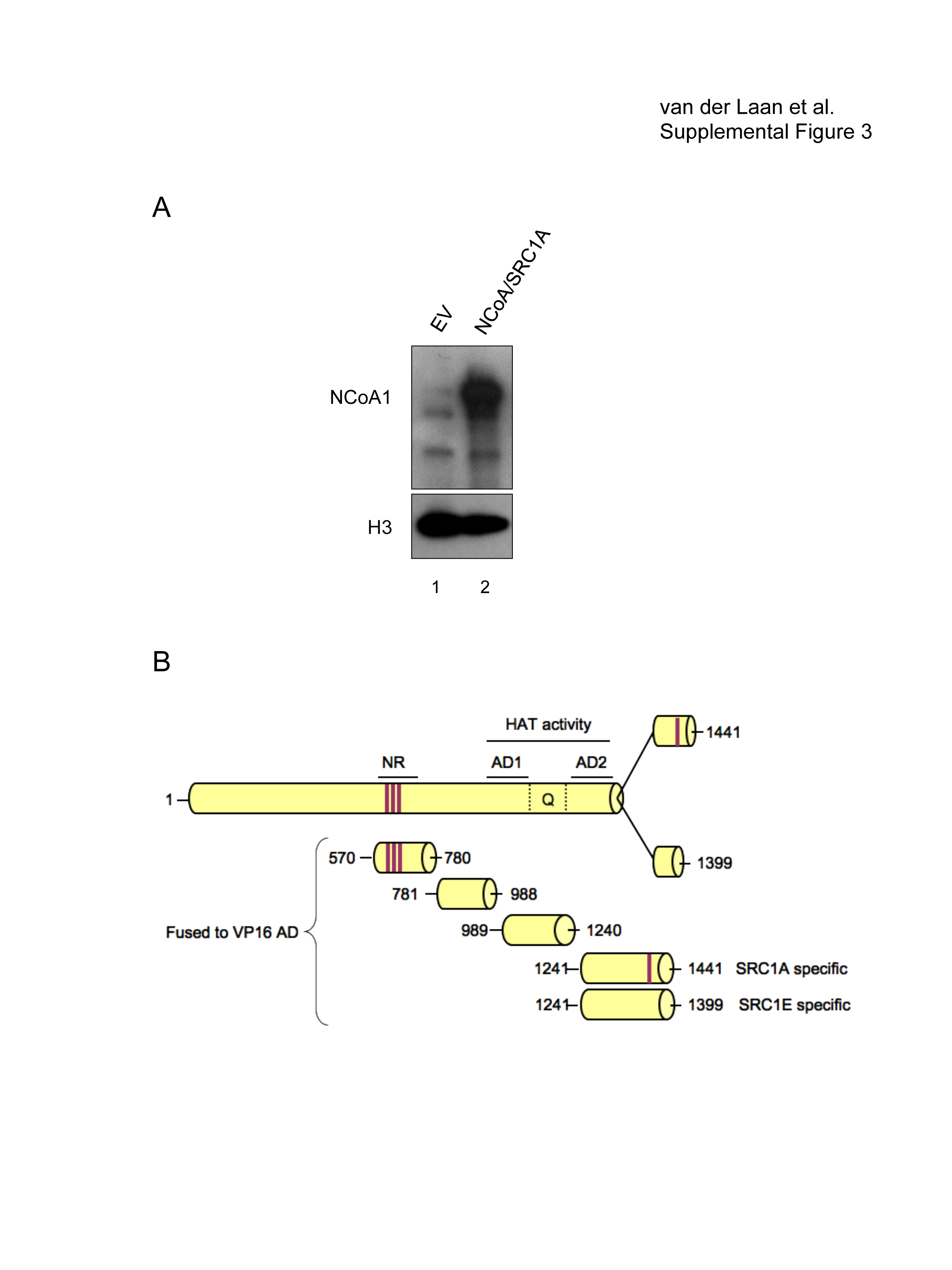

Supplement: Figure S3 — (A) Testing of the SRC1 antibody. Western blot analysis of mESCs transfected with NCoA/SRC1A isoform DNA plasmid. Detection of the Histone H3 is used as loading control. (B) Schematic representation of SRC1 splice variants and VP16 chimeras used for protein-protein interaction assays. The purple bars indicate LxxLL motifs. NR (nuclear receptor box), AD1 (Activation domain 1), AD2 (Activation domain 2) and Q stands for glutamine rich domain. (TIF) [file pone.0093663.s003.tif]
