# Supplementary material for: Cell Cycle-Dependent Expression of Dub3, Nanog and the p160 Family of Nuclear Receptor Coactivators (NCoAs) in Mouse Embryonic Stem Cells
Source: PLoS One. 2014 Apr 2;9(4):e93663. doi: 10.1371/journal.pone.0093663 (PMC3973558; doi:10.1371/journal.pone.0093663)
Supplement: Text S1 — Supporting materials and methods. (DOC) [file pone.0093663.s004.doc]

**Supplemental information**

**Cell Cycle-Dependent Expression of Dub3, Nanog and the p160 family of nuclear receptor coactivators (NCoAs)** **in Mouse ESCs**

Siem VAN DER LAAN^1^, Eleonora GOLFETTO^1^, Jean-Marc VANACKER^2^ and Domenico MAIORANO^1*^

**Supplemental Material & Methods:**

**Fractionation**

CV1 cells were transfected and collected 48 hours post-transfection. Chromatin-enriched and soluble fractions were prepared using CSK-extraction procedure. Briefly, cells were lysed in CSK buffer (10 mM PIPES pH 6.8, 100 mM NaCl, 300 mM sucrose, 1 mM EGTA, 1 mM MgCl_2_, 0.5 mM DTT, 1 mM ATP, 0.2% Triton X-100 and protease inhibitors) All samples were sonicaed at for 5 min with 30” on/off cycles using the Bioruptor (Diagenode). Next, samples were boiled 10 min at 95°C and cooled at room temperature before loading them for western blot analysis.

**Western blotting**

Cells were collected and incubated with ice cold lysis buffer (50 mM Tris-HCl pH 7.4, 100 mM NaCl, 50 mM NaF, 5 mM EDTA, 40 mM β-glycero-phosphate, 1% Triton X-100 and protease inhibitors) for 30 min on ice. Whole cell extracts were clarified by centrifugation at 12000 rcf for 10 min at 4°C. Protein concentration of the clarified lysates was estimated using BCA method (Pierce). Equal amount of protein was used for western blot analysis. All antibodies were incubated overnight at 4°C in phosphate-buffered saline (PBS) containing 1%BSA and 0,1%Tween (Sigma). Signal was detected using Lumi-Light Western Blotting Substrate (Roche; cat. #12015200001) and Kodak film and Amersham Hyperfilm ECL. The following antibodies were used: Sigma, FLAG M2 (F1804); Cell Signaling, Cdk2^Y15P^ (9111), Chk1^S345^ (2341); Abcam, PSTAIR (ab10345), H3(ab1791); Santa Cruz, Cdc25A (sc-7389), HA (sc-805), SRC1(sc-8995), Chk1 (sc-8408).

**Flow cytometry**

Single-cell suspensions of mESC and NIH-3t3 cells were prepared by trypsinisation and washed once in PBS. Cells were fixed in ice-cold 70% ethanol (-20°C) and stored at -20 °C overnight. Following RNAse A treatment, total DNA was stained with propidium iodide (25 µg/ml). For BrdU uptake analysis NIH-3t3 cells were grown in the presence of 10 µM BrdU for respectively 30 minutes. The BrdU content was determined by reaction with a fluorescein isothiocyanate (FITC)-conjugated anti-BrdU antibody (BD Biosciences). Cells were analyzed with a FACScalibur flow cytometer using CellQuestPro software.

Primers used for qPCR

| **Gene name** | **Left primer** | **Right primer** |
| --- | --- | --- |
| Cyclin A2 | cttggctgcaccaacagtaa | caaactcagttctcccaaaaaca |
| Cyclin E1 | ttctgcagcgtcatcctct | tggagcttatagacttcgcaca |
| Nanog | ttcttgcttacaagggtctgc | agaggaagggcgaggaga |
| Oct4 | gttggagaaggtggaaccaa | ctccttctgcagggctttc |
| β-TrCP | gattatggacccggcagag | gagacctgggcatagagcac |
| Cdh1 | cgtgtacctgtggagtgcat | acctactgcgaccaagttcc |
| Dub3 | gctctttccttcccagaagc | gactgtgctttccattggtagtt |
| NCoA1 | tggcatgaacatgaggtcag | gccaacatctgagcattcaa |
| NCoA/SRC1A | gctctcgtccactgaccttc | ctgacgtgggcttttgagtt |
| NCoA/SRC1E | gctctcgtccactgaccttc | tgtagtcaccacagagaagaactc |
| NCoA2 | gccaaggaaacttagggaaca | ctctgtggtgcccattcc |
| NCoA3 | ctggcactgctgtgatgag | agccatttgggcattaaaga |
| Sox2 | ggcagagaagagagtgtttgc | tcttctttctcccagcccta |
| Esrrb | tgcagagtgcctggatggag | ggctcggtaaaggtccagca |
| Noxa | cagatgcctgggaagtcg | tgagcacactcgtccttcaa |
| Chk1 | actgggatttggtgcaaact | cagttattctattcacagcaagttgaa |
| Nestin | tcccttagtctggaagtggcta | ggtgtctgcaagcgagagtt |
| Sdha | tgttcagttccaccccaca | tctccacgacacccttctg |
| Hprt | tcctcctcagaccgctttt | cctggttcatcatcgctaatc |
| Hmbs | tccctgaaggatgtgcctac | aagggttttcccgtttgc |
| β-actin | ctaaggccaaccgtgaaaag | accagaggcatacagggaca |
